# Modified Posterior Trans-olecranon Approach in Tri-vision for Dubberley Type 3B Coronal Shear Fractures of the Distal Humerus

**DOI:** 10.7759/cureus.25175

**Published:** 2022-05-20

**Authors:** Hidetoshi Teraura, Hiroyuki Gotani, Hideki Sakanaka

**Affiliations:** 1 Hand Surgery, Higashisumiyoshi Morimoto Hospital, Osaka, JPN; 2 Hand and Trauma Microsurgery Center, Osaka Ekisaikai Hospital of Japan Seafarers Relief Association, Osaka, JPN; 3 Orthopaedic Surgery, Seikeikai Hospital, Osaka, JPN

**Keywords:** approach, internal fixation, elbow joint, distal humerus fracture, coronal shear fracture

## Abstract

Coronal shear fractures are rare injuries and standard treatment is yet to be determined. There is still no standard approach and fixation method for Dubberley type 3B cases, which are severe fractures that extend to the ulnar side and are accompanied by posterior comminution, making them challenging injuries. We used a modified posterior trans-olecranon approach in tri-vision in the supine position in two type 3B cases. Bone union was achieved in both cases, which exhibited relatively good treatment outcomes with a mean range of motion of -20° for extension and 127.5° for flexion; mean Mayo Elbow performance score of 90; and mean disabilities of the shoulder, arm, and hand score of 20 points. Thus, a modified posterior trans-olecranon approach in tri-vision is useful for type 3B fractures.

## Introduction

Coronal shear fractures (CSF) are rare and there is still no standard treatment [1]. For cases of Dubberley types 1A and 2A [2], which are not severe, good outcomes for osteosynthesis have been reported with an anterolateral or extensile lateral approach, with arthroscopic repair of the fracture using a headless compression screw (HCS) [1,3,4]. However, there is no consensus on the proper approach and fixation method for difficult Dubberley type 3B cases, which are severe fractures that extend to the ulnar side and are accompanied by posterior comminution. An effective approach for type 3B fractures would involve good development from the anterior to posterior surface of the capitellum-trochlea, avoiding excessive traction on the nerves and vascular bundles during osteosynthesis. We have been operating on type 3B cases using a modified posterior trans-olecranon approach in tri-vision in the supine position to develop the anterior, articular, and posterior surfaces of the distal humerus. Here, we report our experience in using the tri-vision approach on two type 3B cases.

## Case presentation

Case 1

**Figure 1:**
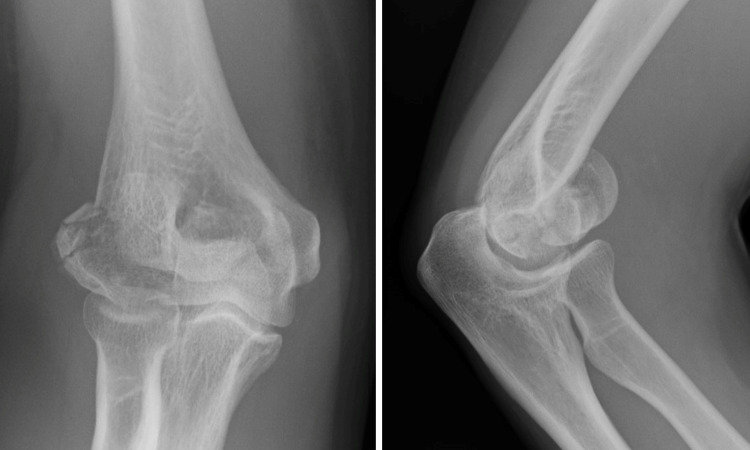
A preoperative radiograph of case 1 showing a fracture from the lateral distal humerus to the trochlea, with a positive double arc sign on the lateral view.

**Figure 2:**
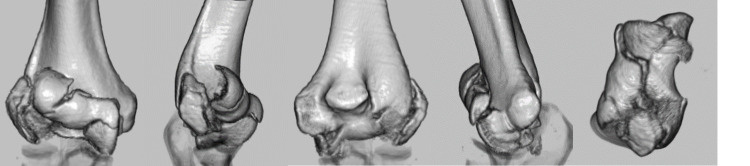
A preoperative three-dimensional computed tomography. Anterior view, radial view, posterior view, ulnar view, and articular surface.

A 57-year-old woman, who injured her right arm in a fall while walking downhill in a park, was brought to our hospital by ambulance. A frontal radiograph upon admission showed a fracture from the lateral distal humerus to the trochlea, with a positive double arc sign on the lateral view (Figure 1). There was a collapse on the lateral side as indicated by a carrying angle of 155° on the injured side, compared with 160° on the uninjured side. Computed tomography (CT) showed multiple bone fragments from the capitellum to the trochlea, accompanied by comminution of the posterior wall, which we diagnosed as Dubberley type 3B (Figure 2). Surgery was performed 15 days after the injury.

**Figure 3:**
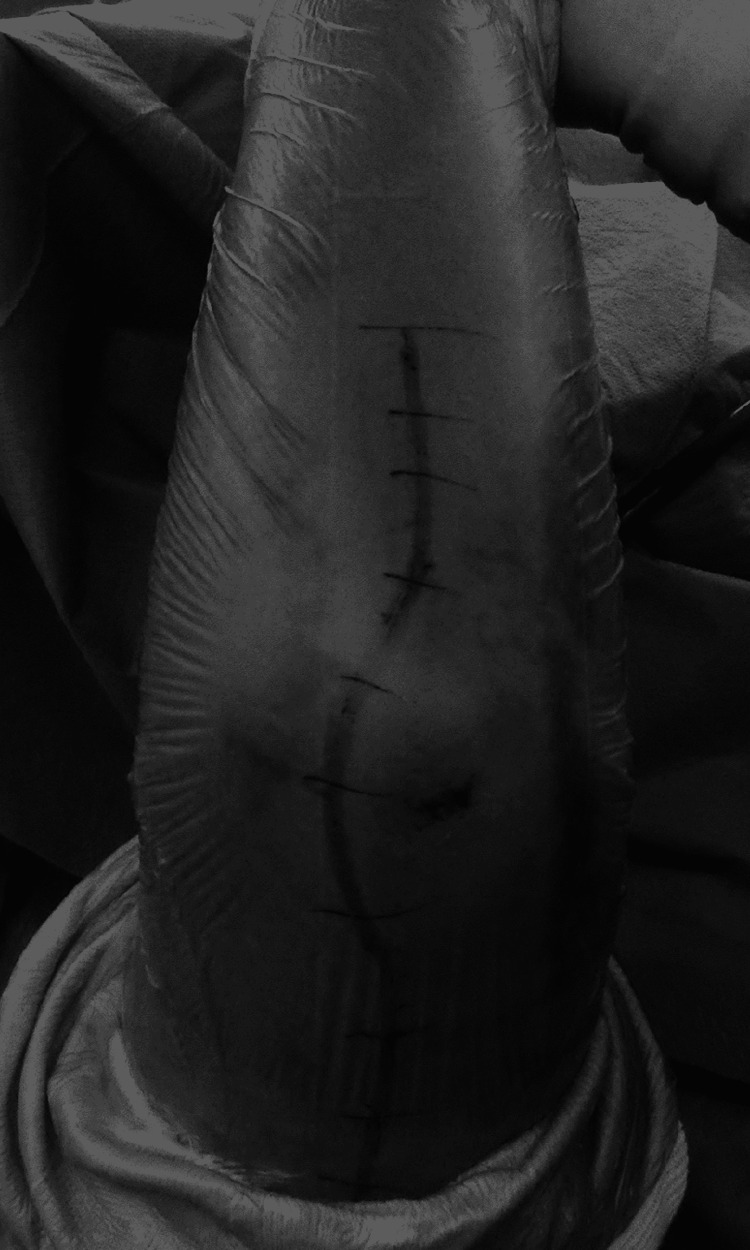
Skin incision. The curved skin incision avoiding the skin just above the olecranon.

**Figure 4:**
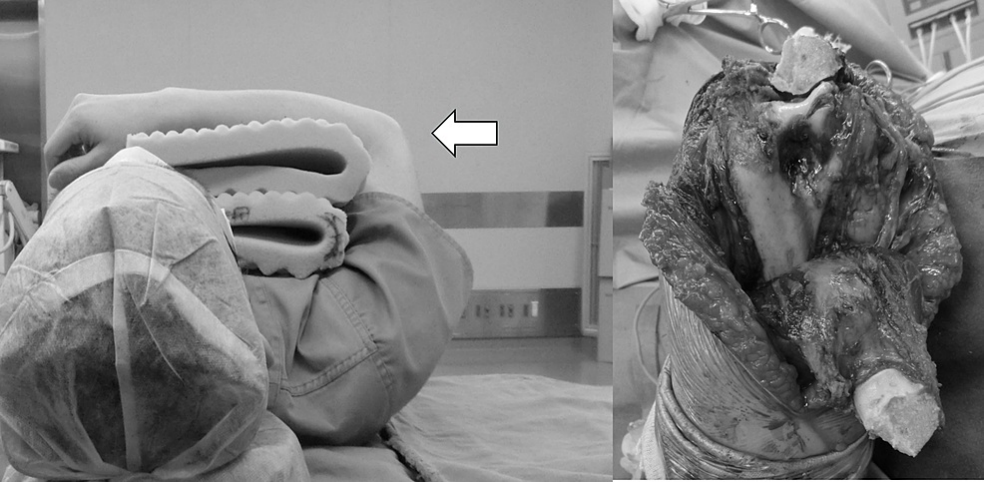
Position and intraoperative photo. Transposing the olecranon proximally together with the triceps brachii, posterior surface can be operated.

**Figure 5:**
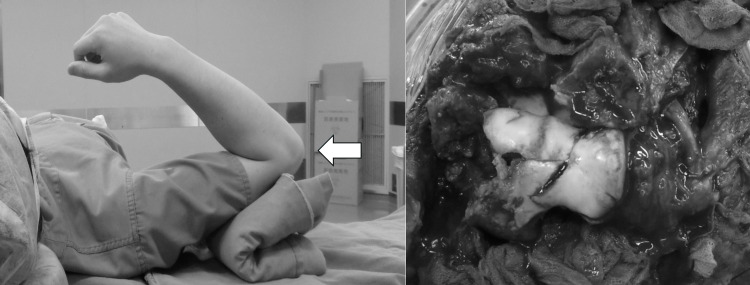
Position and intraoperative photo. Lowering the arm onto a covering cloth on an arm table and flexing the elbow to 120°, articular surface can be operated.

**Figure 6:**
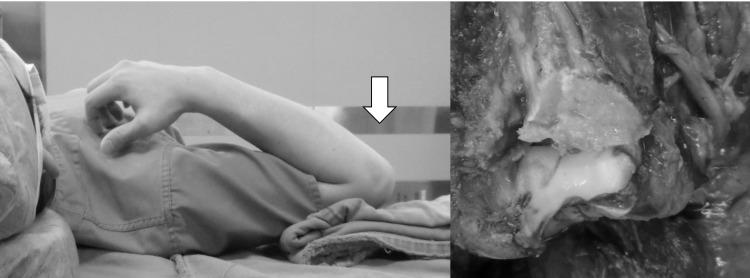
Position and intraoperative photo. Placing the elbow at maximum flexion (140° or more) while being lowered onto an arm table, anterior surface can be operated.

**Figure 7:**
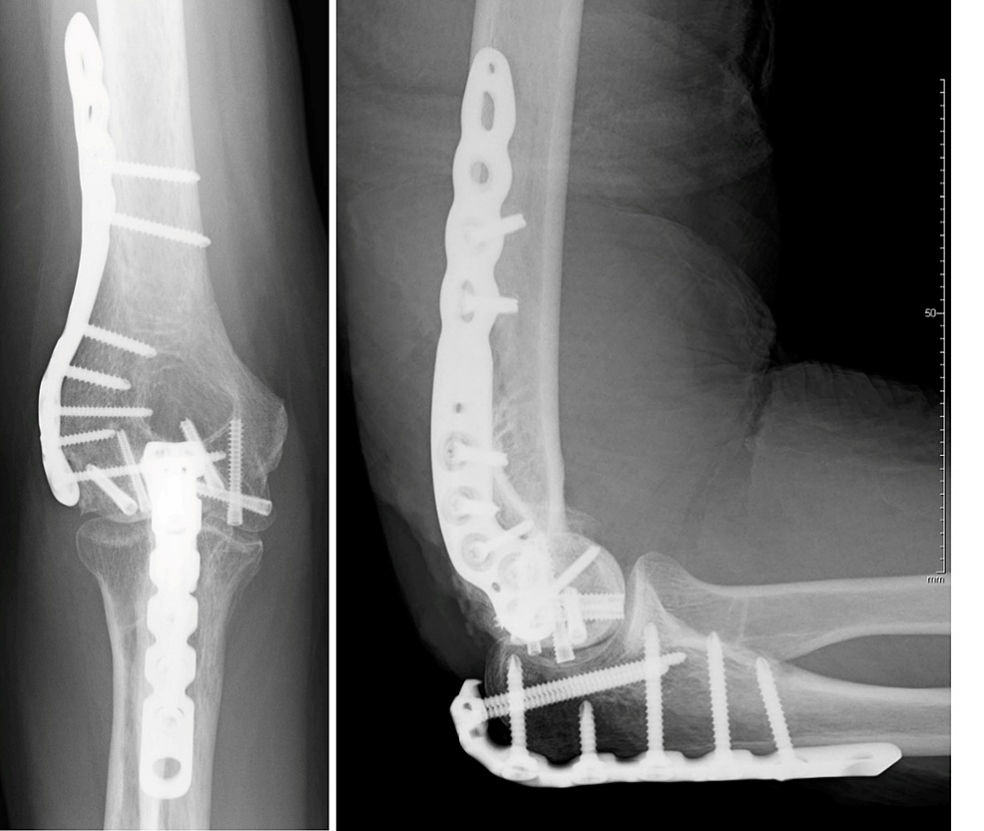
Postoperative radiograph showing obtaining anatomical reduction and stable fixation.

Surgery was performed with the patient in the supine position. First, the arm was raised above the chest with the elbow flexed to 90°. A curved skin incision was made from the posterior forearm proximally to the posterior upper arm (Figure 3). The subcutaneous tissue was dissected to expose the ulnar nerve. The ulnar nerve was detached and placed on the ulnar side so as not to damage it during the procedure. The nerves were not compressed with muscle hooks, and tape was not used to prevent excessive traction. The extensor carpi ulnaris, flexor carpi ulnaris, and anconeus were detached from the proximal ulna to expose the olecranon. V-shaped osteotomy was performed on the olecranon with the Chevron method to transpose the olecranon proximally together with the triceps brachii. This exposed the posterior surface of the distal humerus, allowing the bone fragments to be repaired and fixed (Figure 4). Procedures on the articular surface were performed by lowering the arm onto a covering cloth on an arm table and flexing the elbow to 120° (Figure 5). The manipulations on the anterior surface were performed by placing the elbow at maximum flexion (140° or more) while being lowered onto an arm table. Maximum flexion allowed us to repair and fix the bone fragments in the anterior distal humerus without the forearm bones getting in the way (Figure 6). The collapse of the trochlea and posterior-to-central articular surface was repaired, and artificial bone was used to fill the bone defect. The CSF bone fragments anterior to the capitellum-trochlea were translocated and adhered proximally, and they were detached and repaired. Two ACUTRAK 2 mini (Acumed, Hillsboro, Oregon, USA) were used for the capitellum fragments, while three were used for the trochlear fragments. One ACUTRAK 2 micro was used for the posterior/central articular surface fragments. Because the bone fragments in the posterior lateral condyle were comminuted, they were held down on the surface using the Super FIXORB Mesh (Johnson & Johnson, New Brunswick, New Jersey, USA). On the top of it, an external Variax Elbow Plate (Stryker, Kalamazoo, Michigan, USA) was used to fix the lateral condyle. OSferion 60 (Olympus, Tokyo) was used to fill the bone defect. PERI-LOC Ti Olecranon plate (Smith & Nephew, Watford, UK) was used to fix the olecranon osteotomy area (Figure 7). The ulnar nerve was moved anteriorly and covered with a fat flap. After confirmation of nearly full-range passive range of motion (ROM) with no pressure or cracking sounds, the operation was concluded.

An Ultraflex (Advanfit, Kumamoto, Japan) elbow orthosis was provided 10 days postoperatively to begin ROM practice. The patient reported slight pain 15 months postoperatively, but no joint instability. ROM was as follows: -20° extension, 120° flexion, 85° internal rotation, and 80° external rotation. Radiography showed bone union with no signs of osteonecrosis, pseudoarthrosis, or arthropathic changes. There were no complications such as infection or nerve damage. She had a Mayo Elbow Performance Score (MEPS) of 80 and a Disabilities of the Shoulder, Arm, and Hand (DASH) score of 28.

Case 2

**Figure 8:**
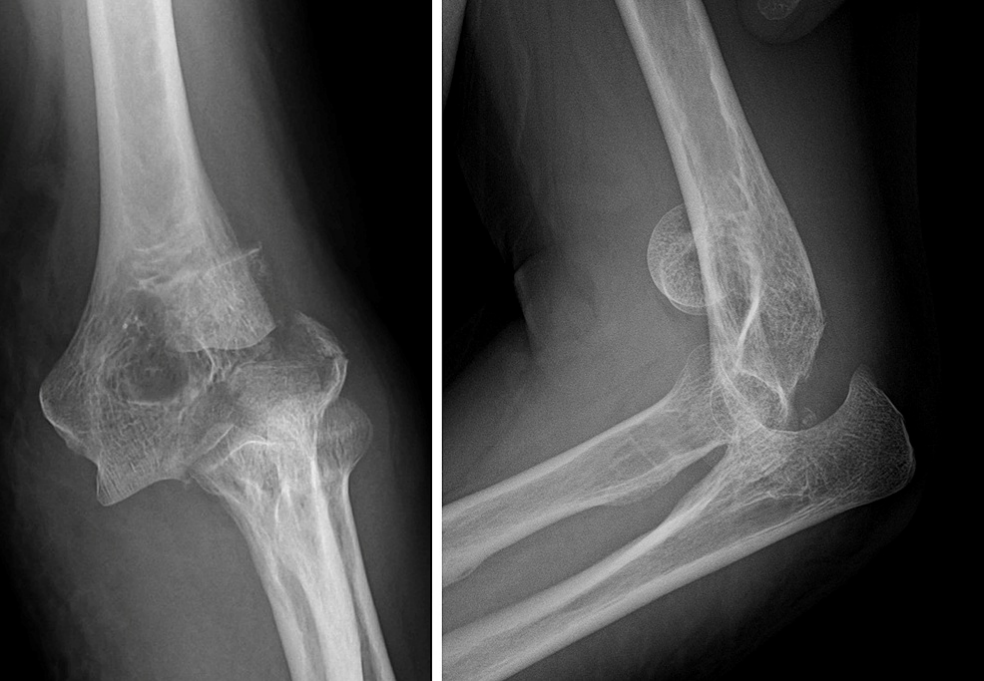
A preoperative radiograph of case 2 showing a fracture from the lateral distal humerus to the trochlea and dislocation to the radial side, with the double arc sign evident on the lateral view.

**Figure 9:**
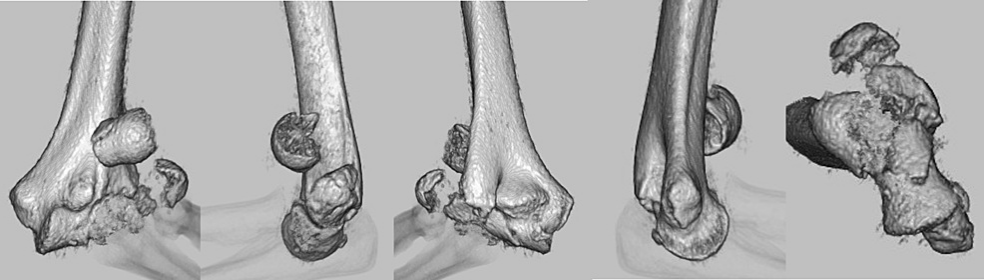
A preoperative three-dimensional computed tomography. Anterior view, radial view, posterior view, ulnar view, and articular surface.

A 71-year-old woman, who was injured by a blow on her left elbow when she fell from her bed at home, was referred to the emergency room of our hospital after she visited a local doctor. A frontal radiograph upon admission showed a fracture from the lateral distal humerus to the trochlea and dislocation to the radial side, with the double arc sign evident on the lateral view (Figure 8). CT showed multiple bone fragments from the lateral condyle of the humerus to the trochlea, with comminution posterior to the lateral condyle. The diagnosis was Dubberley type 3B (Figure 9). Surgery was performed five days after the injury. Similar to Case 1, the surgery was performed with the patient in the supine position with the modified posterior trans-olecranon approach in tri-vision.

**Figure 10:**
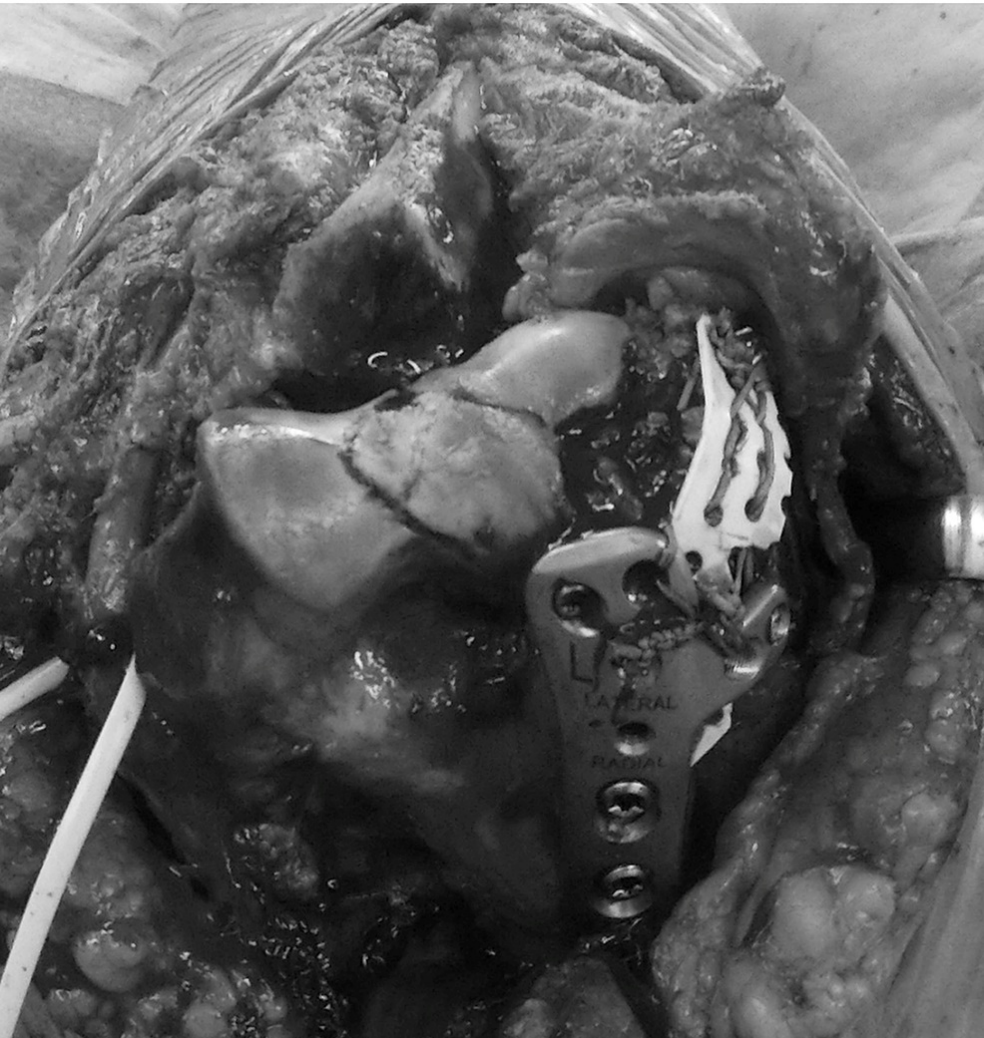
Intraoperative photo. The extensor muscles and Super FIXORB Mesh were sutured to the plate with non-absorbent thread.

**Figure 11:**
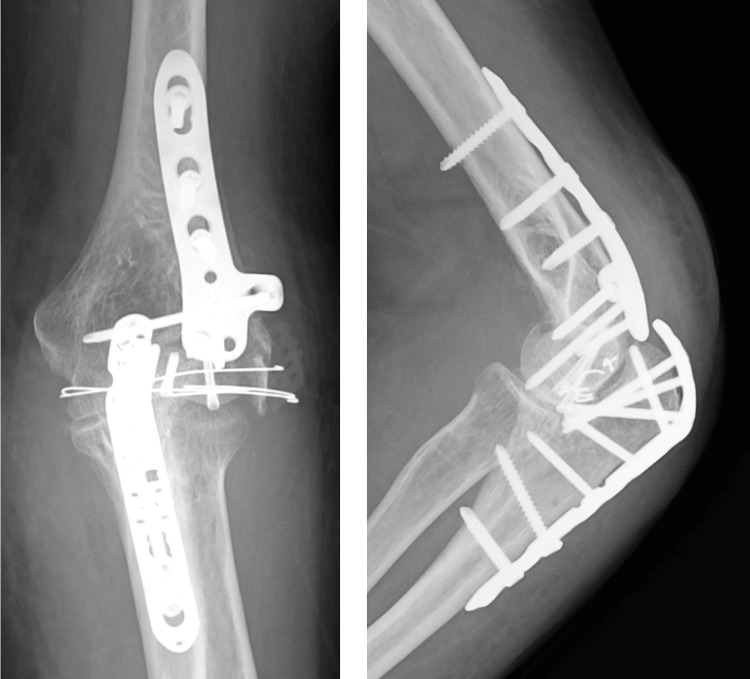
Postoperative radiograph showing obtaining anatomical reduction and stable fixation.

The collapsed trochlea was elevated and repaired with two 0.7-mm Kirschner wires. Because of the proximal translocation of the capitate fragment, it was repaired and fixed to the trochlear fragment using two ACUTRAK 2 micro (Acumed, Hillsboro, Oregon, USA). The lateral epicondyle was repaired together with the extensor tendon attachment. The comminuted bone fragments of the posterior lateral condyle were held down on the surface using Super FIXORB Mesh (Johnson & Johnson, New Brunswick, New Jersey, USA). On the top of it, an LCP DISTAL HUMERUS PLATE with lateral support (three holes) (DePuy Synthes, New Brunswick, New Jersey, USA) was fixed. Two distal locking screws were inserted. One was used to posteriorly fix the trochlear and capitate fragments. The extensor muscles and Super FIXORB Mesh were sutured to the plate with ULTRA BRAID (Smith & Nephew, Watford, UK) (Figure 10). Because the lateral epicondyle fragment was still unstable, three additional 0.7-mm Kirschner wires were inserted. The olecranon osteotomy site was fixed with a VA LCP Proximal Olecranon Plate (DePuy Synthes, New Brunswick, New Jersey, USA) (Figure 11). The medial collateral ligament was repaired using a PANALOK anchor (DePuy Mitek, New Brunswick, New Jersey, USA). The ulnar nerve was moved anteriorly and covered with a fat flap. After confirmation of nearly full-range passive range of motion (ROM) with no pressure or cracking sounds, the operation was concluded.

**Figure 12:**
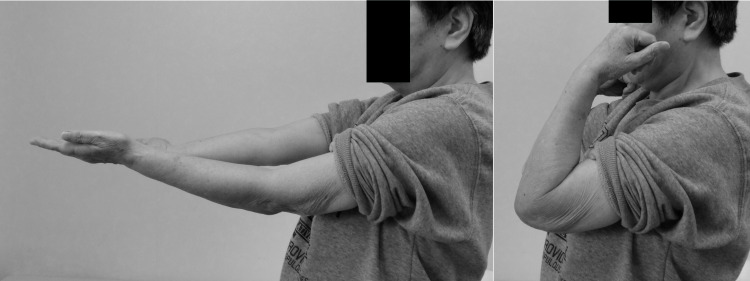
The image of flexion and extension at 14 months postoperatively.

**Figure 13:**
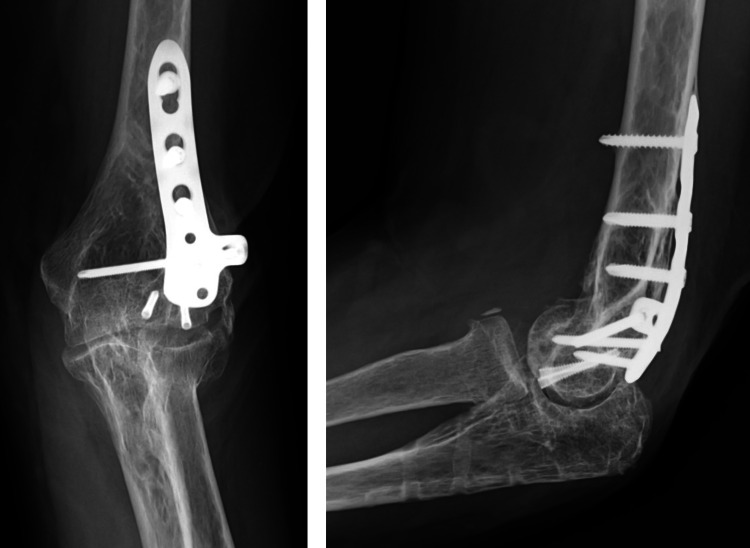
Radiograph at 14 months postoperatively showing that bone union had been achieved. There was no osteonecrosis, but mild arthropathic changes were observed.

An Ultraflex elbow orthosis was provided on postoperative day 7 to begin ROM practice. At 14 months postoperatively, the patient reported no pain or joint instability. ROM was as follows: -20° extension, 135° flexion, 85° internal rotation, and 90° external rotation (Figure 12). Radiography showed that bone union had been achieved. There was no osteonecrosis, but mild arthropathic changes of Broberg and Morrey radiographic grade I [5] were observed (Figure 13). There were no complications such as infection or nerve damage. She had a MEPS of 100 and a DASH score of 12.5.

## Discussion

In 1996, McKee et al. reported a humeral capitellum fracture in which a shearing force created a fracture line in the frontal plane direction extending from the capitellum to the trochlea, which they called a CSF [6]. This type of fracture makes up 6% of distal humeral fractures and 1% of fractures around the elbow [1]. Being an intra-articular fracture, it requires precise repair and firm fixation, and it is important to select the correct approach and fixation method for this fracture type. The greater the extension of the fracture to the ulnar side and severity of comminution, the greater the difficulty for treatment. In 2006, Dubberley et al. reported classifications that considered the extent of the fracture and the degree of comminution [2]. These classifications reflect the severity and thus help guide treatment. Evaluating fractures with only simple radiography is difficult, and CT, especially 3D-CT, is more useful [7].

Reports on this type of fracture indicate that greater severity is associated with poorer outcomes. In a report on 52 cases by Song et al. [8], overall outcomes were good, with a mean MEPS of 90.6; excellent in 36 cases; good in 11 cases; and fair in five cases, with ROM extension at 3° and flexion at 136°. However, all four cases of type 3B were fair. They also reported that MEPS was significantly different between cases with (71.4) and without (93.6) posterior comminution. Regarding pseudarthrosis, Brouwer et al. [9] reported that type 3B had a higher pseudarthrosis rate than other fracture types, with pseudarthrosis observed in eight of 18 cases (44%). According to Marinelli et al. [10], the MEPSs of type A and type B cases were 86 and 73, respectively. Type A had significantly better ROM (64%) and type B had significantly more complications (29%), with poorer treatment outcomes in cases with posterior comminution.

The principles of treatment are to regain anatomical joint compatibility, firmly fix the fracture, and start early training to improve ROM. Treatment results in good outcomes, despite its difficulty. The difficulties in developing the entire fractured part and fixation are the two reasons for the complicated treatment. An approach is needed that can develop the posterior, articular, and anterior surfaces of the distal humerus for repair and fixation while avoiding excessive traction on the nerves or blood vessels. Ring et al. reported ulnar neuropathy in two of 21 cases that required anterior subcutaneous transposition [11]. Various approaches for coronal shear fractures have been reported. There is the extensile lateral approach [11,12], when the fracture is limited to the lateral side, or the anterolateral approach [13-15], when the fracture includes the capitellum and trochlea. However, repairing and fixing type 3B fractures is difficult with these procedures. This type requires olecranon osteotomy, which allows for the anterior, articular, and posterior surfaces of the distal humerus to be developed and manipulated [2,12]. We also use olecranon osteotomy to develop the fracture areas, but we perform the procedure with the patient in the supine position instead of the typical prone or lateral positions [16]. In our supine/olecranon osteotomy approach, osteotomy and transposition of the olecranon allow the development and manipulation of the posterior surface of the distal humerus. In the supine position, the elbow can be placed in maximum flexion, which allows for the development and manipulation of the articular and anterior surfaces of the distal humerus (Figure 6). The ulna, which is usually an obstacle when manipulating the anterior surface of the distal end of the humerus, is no longer in the way. Repairing bone fragments can be done under direct visualization, and drill screws can be inserted in any direction. There is also almost no possibility of placing traction on the nerves and there is no need to dissect the lateral collateral ligament.

The fracture needs to be fixed to withstand early movement, but the difficulty of the fixation method makes the selection of an implant challenging. It has been agreed that large fragments of the capitellum should be fixed with HCS such as ACUTRAK screws. Depending on the fracture type, threaded Kirschner wires, locking plates, anti-glide plates, bioabsorbable implants, and other tools can also be used [7,12,17]. The authors also use various implants depending on the fracture type. For small bone fragments that are difficult to fix with screws, SUPER FIXORB Mesh is used to hold them on the surface. A plate is then fixed on the top (Figure 10).

Starting ROM training early is best for postoperative aftercare. In our department, after about one week of external immobilization, we start ROM practice with the patient wearing an Ultraflex orthosis with bilateral columns and gear and lock mechanisms. For the first week, the training is limited to 60° extension and 120° flexion (60° arc). After that, gradually increase the angle of gear and the lock mechanisms. The bilateral columns reduce lateral stress during training, and the gears allow the patient to practice passive ROM independently.

## Conclusions

Dubberley type 3B CSF of the distal humerus is challenging to treat owing to the difficulty of repairing and fixing the fracture. Our modified posterior trans-olecranon approach in tri-vision in the supine position allows the development and fixation of the entire fracture, without the nerves or vascular bundles getting in the way or causing postoperative nerve paralysis. Strong fixation can be obtained with various implants, such as HCS or locking plates, depending on the situation. Good treatment outcomes were achieved with postoperative orthosis therapy. Improving treatment outcomes requires an approach, implant, and fixation method suitable to the fracture type, along with appropriate postoperative therapy.
